# Malingering under the Old Regime

**Published:** 1888-09-15

**Authors:** W. Curran

**Affiliations:** A.M.D.


### Malingering under the Old Regime.

By W. Curran, A.M.D.

Having recovered my lost, or rather mislaid, copy of Marshall's erewhile famous book on "The Enlisting, Discharging, and Pensioning of Soldiers," I am induced to extract a few items from it, and thereby show the prevalence, etc., of this practice under the old *régime* of cruel punishments, vexatious enactments, and interminable military service. Numberless were the devices or dodges that the soldier resorted to in those evil days, and equally numerous were the recipes of nostrums he employed for effecting his purpose. These are given here, and they include such substances as lime, copperas, corrosive sublimate, tobacco, ashes, etc. Self-mutilation was frequently thrown in.

He carried about with him instructions for injuring an eye, simultating a rupture, or exciting an ulcer, and his endurance under treatment—oh! such treatment—was worthy of a Christian martyr, or of an Indian brave. His diet was very meagre and most disrelishing in hospital, and the outward applications or inward remedies he was subjected to were such as would disgust a starving dog, or extract groans from—imoa pec tore—a dying camel. He was bled and leeched till his complexion resembled that of a wax doll or a marble statue; his head reeled under this strain, and frightful dreams or hallucinations disturbed his night's repose.

Yet did he persevere, and often won by this dogged endurance the object of his depraved ambition. He wore out, in short, the patience of his superiors, and there are, it must be confessed, some real and not unimportant diseases which are not indicated by any change of the pulse, by any remarkable alteration of the colour or temperature of the body, or by any evident derangement of its functions. The clinical thermometer was unknown in those days, so was that all potent detector of stiff knees or immovable elbow joints chloroform; and there is no doubt that diseases, which were at first supposed to be feigned, have eventually proved to be genuine. The medical officer had, therefore, to be on his guard. He stood, as it were, between two fires—that of his own professional repute or conscience on the one hand, and that of his irate colonel on the other; and he knew that however much appearances might be against his patient, he—the surgeon—had to proceed regularly, deliberately, and patiently in every case. It was, in a word, a game of endurance between the determined malingerer and the doctor, and our author confesses that "old soldiers who prosecute their schemes with art—who possess great fortitude and an inflexible resolution—will commonly succeed in getting their discharge, either by making falsehood appear to be more probable than truth, or perhaps more frequently by exhausting the patience of medical and commanding officers."

Dr. Marshall gives many illustrations of these morbid fancies or factitious ailments, and some of these are so curious as to read more like the revelations of Miss Braddon or Mr. Wilkie Collins, than the quasi official statement of a sober military surgeon of the old school. I will quote a few of these, and then close this portion of my thesis. "A recruit of those days . . . alleged that he had almost totally lost the sense of hearing (and it may be here incidentally observed that a simulator of deafness is rarely discovered), and the testimony of his comrades served to support his statements. The surgeon of the depot admitted him to hospital, and fed him on spoon diet. He purposely passed his bed during his daily visits for nine days without noticing him. On the tenth day he felt his pulse, and made signs to him to put out his tongue; he then asked the hospital sergeant what diet he gave him. 'Spoon diet' replied the sergeant. The doctor affected to be displeased, and in a low tone said, 'Are you not ashamed of yourself? the poor fellow is almost starved to death. Let him instantly have a beef steak and a pint of porter.' The recruit could contain himself no longer. With a countenance expressive of gladness and gratitude, he said 'God Almighty bless your honour; you are the best gentleman I have seen for many a day,'" and several other similar stories are narrated here.

"Two soldiers were transferred from a regiment in Ireland to the General Hospital, Dublin, for the purpose of being discharged, in consequence of alleged incurable disabilities. The countenance and general appearance of the men indicated little, if any, derangment of the healthy functions of the body. The medical officer made them stand up together, and requested one of them to give an account of his complaint, which he did very fully, being encouraged to enter minutely into a detail of symptoms and feelings. By his account it appeared that he suffered more or less under disease from the crown of the head to the sole of the foot. Not a word indicative of incredulity on the part of the surgeon was uttered in regard to the man's statement. The other man was then asked: 'What is the matter with you?' 'The same as this man,' was his ready reply. The inference was (our author observes) obvious, he was evidently not prepared with a set of symptoms, and without perceiving the trap laid for him, he instinctively availed himself of the talent and ingenuity of his fellow impostor. They were both sent back to their respective regiments, and Dr. Marshall had no doubt that each of them ultimately succeeded in 'working out' a discharge.

"Private J. W. was sent home from India for the purpose of being invalided at Fort Pitt. He stooped so much that the upper part of his body formed nearly a right angle with his inferior extremities, and he usually moved from place to place by the help of a stick about two feet long, which he grasped by the middle. This disability being presumed to be feigned, he was placed on his back on the floor, when he held his legs in a nearly vertical direction, and complained piteously at the attempts that were made to straighten them. The position was reversed and he supported himself for some time on his head, hands, and feet. He soon became tired of this position, and at last, when he could endure it no longer, he stretched his legs fully out and lay flat upon his chest and stomach. He then warmly expressed his gratitude to the medical officer for having so effectually cured him of a disability under which he had long suffered." And we may thank short service, the abolition of flogging, etc., for the fact that scenes of this kind are no longer possible.

The following case—with which I will close this series—illustrates as well the endurance of these worthies as it does the success that occasionally crowned their efforts, and Dr. Marshall truly remarks, in connexion with it, that "paraplegia—the disqualification here counterfeited—is sometimes simulated, and the most uncomfortable condition endured with martyr-like perseverance." "A man belonging to the Cavan Militia alleged that he had lost the power of his inferior extremities, and was recommended to be discharged on account of palsy. Having succeeded after much torture and misery in getting this, as well as the balance of his pay, he hired a car, and was driven in it to the Phœnix Park, where his regiment was at the time at exercise. Upon approaching the corps, he threw away his crutches, and bounded like a deer for some time in front of the line. After slapping his thighs, he scampered off as fast as he could," and it may be doubted, after all, whether he was not, under the circumstances, a good riddance.

Doubtless malingering still goes on, but it is now in a modified form. Short service may have its defects, but at least it encourages the soldier to endure the *désagrémens* inseparable from his life with patience, because he knows they will not last for ever.

